# An Extended Surface Loop on *Toxoplasma gondii* Apical Membrane Antigen 1 (AMA1) Governs Ligand Binding Selectivity

**DOI:** 10.1371/journal.pone.0126206

**Published:** 2015-05-08

**Authors:** Michelle L. Parker, Martin J. Boulanger

**Affiliations:** Department of Biochemistry & Microbiology, University of Victoria, PO Box 3055 STN CSC, Victoria, BC, V8W 3P6, Canada; University at Buffalo, UNITED STATES

## Abstract

Apicomplexan parasites are the causative agents of globally prevalent diseases including malaria and toxoplasmosis. These obligate intracellular pathogens have evolved a sophisticated host cell invasion strategy that relies on a parasite-host cell junction anchored by interactions between apical membrane antigens (AMAs) on the parasite surface and rhoptry neck 2 (RON2) proteins discharged from the parasite and embedded in the host cell membrane. Key to formation of the AMA1-RON2 complex is displacement of an extended surface loop on AMA1 called the DII loop. While conformational flexibility of the DII loop is required to expose the mature RON2 binding groove, a definitive role of this substructure has not been elucidated. To establish a role of the DII loop in *Toxoplasma gondii* AMA1, we engineered a form of the protein where the mobile portion of the loop was replaced with a short Gly-Ser linker (*Tg*AMA1ΔDIIloop). Isothermal titration calorimetry measurements with a panel of RON2 peptides revealed an influential role for the DII loop in governing selectivity. Most notably, an *Eimeria tenella* RON2 (*Et*RON2) peptide that showed only weak binding to *Tg*AMA1 bound with high affinity to *Tg*AMA1ΔDIIloop. To define the molecular basis for the differential binding, we determined the crystal structure of *Tg*AMA1ΔDIIloop in complex with the *Et*RON2 peptide. When analyzed in the context of existing AMA1-RON2 structures, spatially distinct anchor points in the AMA1 groove were identified that, when engaged, appear to provide the necessary traction to outcompete the DII loop. Collectively, these data support a model where the AMA1 DII loop serves as a structural gatekeeper to selectively filter out ligands otherwise capable of binding with high affinity in the AMA1 apical groove. These data also highlight the importance of considering the functional implications of the DII loop in the ongoing development of therapeutic intervention strategies targeting the AMA1-RON2 invasion complex.

## Introduction

Parasites of phylum Apicomplexa cause devastating diseases on a global scale. Species within the *Plasmodium*, *Toxoplasma*, and *Eimeria* genera, for example, are the etiological agents of malaria, toxoplasmosis, and coccidiosis, respectively. *P*. *falciparum* causes the most severe cases of human malaria [1], while *T*. *gondii* infects up to a third of the world’s human population and leads to a significant number of premature abortions in livestock [2], and *E*. *tenella* infections in chickens present an economic burden to the poultry industry estimated to exceed 1.5 billion dollars annually [3]. The widespread success of the apicomplexans relies on a conserved mechanism of host cell invasion, since access to the host cell interior is crucial for survival of these obligate intracellular parasites.

Apicomplexan parasites invade host cells using a highly orchestrated, step-wise mechanism. A parasite initially glides along cell surfaces, followed by reorientation and formation of a tight adhesion between the apical end of the parasite and the target host cell membrane. Subsequently, a circumferential ring of adhesion, termed the moving junction or tight junction, is formed through which the parasite propels itself while concurrently depressing the host cell membrane to form a nascent vacuole essential for parasite survival and replication [4, 5]. Supporting these invasion events are binary complexes formed between the apical membrane antigen (AMA) and rhoptry neck protein 2 (RON2) families that anchor the junction between parasite and host cell [6–10]. Specifically, RON2s are transmembrane proteins secreted by apicomplexans into the host cell, integrated into the outer membrane, and presented on the host cell surface to serve as ligands for AMA proteins on the parasite surface [7, 8, 11, 12]. The number of AMA and RON2 family members capable of forming functional invasion complexes vary across apicomplexans. For example, *Plasmodium spp*. harbour only a single copy of an AMA (AMA1) and RON2, while *T*. *gondii* and other *Eimeriorina*, such as *E*. *tenella*, have four AMAs (AMA1-4) and at least three RON2s (RON2, RON2_L1_, RON2_L2_) that show distinct and often stage-specific expression and interaction patterns [8, 13–16]. In *T*. *gondii*, AMA1 and AMA2 interact with RON2 in tachyzoites, AMA3-RON2_L2_ functions in sporozoites, and the recently identified AMA4-RON2_L1_ pairing is also predicted to play a role in sporozoites [7, 14, 15]. The expression of multiple AMA1 and RON2 paralogues within a species reflects the importance of this pairing to ensuring successful invasion.

Very limited cross-reactivity between AMA and RON2 proteins has been observed [17], yet the mechanisms that underlie this exquisite specificity are only partially understood. It is clear, however, that co-evolution between receptor and ligand plays a role in the selective AMA-RON2 binding events as illustrated by the structural and biochemical characterization of AMA proteins with the corresponding binding region of RON2 (RON2 domain 3; RON2D3) from *T*. *gondii* tachyzoites (AMA1-RON2D3) [18], *T*. *gondii* sporozoites (AMA3-RON2_L2_D3) [15], and *P*. *falciparum* merozoites (AMA1-RON2D3) [19]. The crystal structures of these AMA1-RON2 and AMA3-RON2_L2_ complexes revealed that conformational changes in the apical surface of the AMA1/AMA3 protein are necessary to form a functional RON2 binding groove. In particular, a domain II loop (DII loop) of AMA1/AMA3 undergoes a substantial conformational change to reveal approximately half of the RON2 binding region. While several different roles have been proposed for the DII loop, including signalling associated with moving junction formation, regulating proteolytic processing of AMA1/AMA3, aiding in host immune evasion, and selectively filtering potential binding partners, a definitive role has yet to be elucidated [15, 18, 20–22]. Moreover, the differing length, composition, and flexibility of the DII loop across apicomplexan AMAs leads to highly divergent conformations that suggest the potential for genus-specific functions [17, 23–27].

To investigate the role of the *T*. *gondii* AMA1 DII loop, we engineered a construct of *Tg*AMA1 lacking the flexible portion of the DII loop (*Tg*AMA1ΔDIIloop) and compared it with *Tg*AMA1 for the ability to coordinate a diverse panel of RON2 peptides. Intriguingly, DII loop dependent differences in RON2 coordination were observed. Most notably, an *E*. *tenella* RON2 peptide (*Et*RON2D3) showed significantly tighter binding to *Tg*AMA1ΔDIIloop relative to *Tg*AMA1. Subsequent structural characterization of *Tg*AMA1ΔDIIloop in complex with *Et*RON2D3 provides the first high-resolution view of a cross-genus AMA1-RON2 complex and yields important insight into the AMA1-RON2 coordination event. Collectively, these data reveal a gatekeeper role for the DII loop as it selectively filters access to the AMA1 groove, ensuring exquisite specificity and regulating assembly of the AMA1-RON2 invasion complex.

## Materials and Methods

### Cloning, protein production and purification

A sequence encoding the mature ectoplasmic region of *Tg*AMA1 (amino acids 64 to 484, TGME49_255260 [24]) with the twenty residues of the DII loop disordered in the *Tg*AMA1-*Tg*RON2D3 co-structure [18] replaced by a seven residue Gly-Ser linker (*Tg*AMA1ΔDIIloop) was synthesized and subcloned into a modified pAcGP67b vector (Pharmingen) with a C-terminal thrombin cleavage site and hexahistidine tag. *Tg*AMA1ΔDIIloop encoding virus for insect cell protein production was generated and amplified using established protocols [18, 24]. Following a 65 hr infection the supernatant was harvested, concentrated, and *Tg*AMA1ΔDIIloop was purified by nickel batch binding as previously described [17]. The hexahistidine tag was removed by thrombin cleavage and the protein was further purified by size exclusion chromatography (SEC) (Superdex 16/60 75) in HEPES buffered saline (HBS: 20 mM HEPES pH 7.5, 150 mM NaCl).

A sequence encoding a portion of *Et*RON2D3 (amino acids 1262 to 1297, ETH_00012760) was synthesized and subcloned into a modified pET32a vector (Novagen) incorporating N-terminal hexahistidine and thioredoxin (TRX) tags with a thrombin cleavage site. The fusion protein was produced in *E*. *coli* BL21 cells. For crystallization, *Et*RON2D3-TRX was cleaved to remove the fusion tags and co-purified with *Tg*AMA1ΔDIIloop by SEC in HBS followed by anion exchange chromatography using a Source 30Q column (20 mM HEPES pH 8.0, 10 mM NaCl; elution gradient with 20 mM HEPES pH 8.0, 1 M NaCl) to remove residual TRX tag.

For ITC experiments, *Tg*AMA1 was produced and purified as previously reported [24]. Thioredoxin fusions of *Tg*RON2D3, *Tg*RON2_L1_D3, *Tg*RON2_L2_D3, *Et*RON2D3 and *Pf*RON2D3 were produced in *E*. *coli* BL21 cells and purified by nickel-affinity and SEC.

### Isothermal Titration Calorimetry

Purified *Tg*AMA1, *Tg*AMA1ΔDIIloop, and TRX fusions of *Tg*RON2D3, *Tg*RON2_L1_D3 *Tg*RON2_L2_D3, *Et*RON2D3, and *Pf*RON2D3 were dialyzed against HBS at 4 °C. All ITC experiments were carried out at 25 °C on a MicroCal iTC200 instrument (GE Healthcare). The sample cell contained *Tg*AMA1 or *Tg*AMA1ΔDIIloop (10 to 16 μM for nanomolar affinity, 35 to 75 μM for micromolar affinity), and the TRX-fused peptides (110 to 180 μM for nanomolar affinity, 360 to 1180 μM for micromolar affinity) were added in 17 injections of 2 μL each. TRX was injected as a negative control and showed no detectable binding. Data were processed using Origin software (MicroCal) and the dissociation constants (K_d_) were determined using a one-site model. Values are derived from a single experiment, but are representative of at least two independent experiments.

### Native gel electrophoresis assay

Purified *Tg*AMA1ΔDIIloop and co-purified *Tg*AMA1ΔDIIloop-*Et*RON2D3 protein samples in HBS were run on an 8–25% gradient native gel using the PhastGel system (GE Healthcare), and protein bands were visualized with Coomassie Brilliant Blue staining.

### Crystallization and data collection

Using a Crystal Gryphon (Art Robbins Instruments), crystals of *Tg*AMA1ΔDIIloop were grown in 5 mM cobalt (II) chloride hexahydrate, 5 mM nickel (II) chloride hexahydrate, 5 mM cadmium chloride hydrate, 5 mM magnesium chloride hexahydrate, 0.1 M HEPES pH 7.5, 12% PEG 3350, and 2% glycerol. The final drops for *Tg*AMA1ΔDIIloop consisted of 0.3 μL protein (14 mg/mL) with 0.6 μL reservoir solution and were equilibrated against 55 μL of reservoir solution. Cryoprotection of the *Tg*AMA1ΔDIIloop crystals was carried out in reservoir solution supplemented with 25% glycerol for 20 seconds and the crystals were flash cooled at 100 K directly in the cryo stream.

Using the sitting drop method, crystals of *Tg*AMA1ΔDIIloop-*Et*RON2D3 were grown in 0.1 M tri-sodium citrate pH 5.0 and 2.0 M ammonium sulfate. The final drops for *Tg*AMA1ΔDIIloop-*Et*RON2D3 consisted of 1.2 μL protein (15 mg/mL) with 1.8 μL reservoir solution and were equilibrated against 100 μL of reservoir solution. Cryoprotection of the *Tg*AMA1ΔDIIloop-*Et*RON2D3 crystals was carried out in a 3:1 ratio of 2.5 M lithium sulfate: 1.0 M sodium sulfate for 20 seconds and the crystals were flash cooled at 100 K directly in the cryo stream. All diffraction data were collected on beamline 12–2 at the Stanford Synchrotron Radiation Lightsource (SSRL).

### Data processing, structure solution and refinement

Diffraction data were processed using Imosflm [28] and Scala [29] in the CCP4 suite of programs [30]. Initial phases for both *Tg*AMA1ΔDIIloop and *Tg*AMA1ΔDIIloop-*Et*RON2D3 were obtained by molecular replacement using PHASER [31] with *Tg*AMA1 DI-DII-DIII from the complex with *Tg*RON2D3 (PDB ID: 2Y8T). Solvent molecules were selected using COOT [32] and refinement carried out using Phenix.refine [33]. Structural validation was performed with Molprobity [34]. Overall, 5% of the reflections were set aside for calculation of R_free_. Data collection and refinement statistics are presented in Table 1. The atomic coordinates and structure factors have been deposited in the Protein Data Bank: *Tg*AMA1ΔDIIloop (PDB ID: 4YIV); *Tg*AMA1ΔDIIloop-*Et*RON2D3 (PDB ID: 4YIZ).

**Table 1:** Data collection and refinement statistics.

	*Tg*AMA1ΔDIIloop	*Tg*AMA1ΔDIIloop-*Et*RON2D3
A. Data collection statistics		
Spacegroup	P4_1/3_2_1_2	P3_1/2_2
a, b, c (Å)	89.05, 89.05, 124.97	265.93, 265.93, 94.16
α, β, γ (deg.)	90, 90, 90	90, 90, 120
Wavelength	0.9795	0.9795
Resolution range (Å)	56.24–1.93 (2.11–1.93)	72.89–2.20 (2.32–2.20)
Measured reflections	297853 (43802)	782702 (112655)
Unique reflections	38493 (5516)	180412 (26362)
Redundancy	7.7 (7.9)	4.3 (4.3)
Completeness (%)	99.8 (91.9)	93.9 (94.4)
*I/σ(I)*	13.8 (4.0)	6.6 (3.4)
R_merge_ ^a^	0.081 (0.494)	0.133 (0.475)
B. Refinement statistics		
Spacegroup	P4_1_2_1_2	P3_2_2
Resolution (Å)	51.15–1.93	59.50–2.20
R_work_ ^b^/R_free_ ^c^	0.176/0.195	0.187/0.207
No. of atoms		
Protein (A/B/C/D/E/F)	2950	3119/271/3099/271/3061/271
Sulfate	N/A	25
Co/Cd/Cl	3/2/2	N/A
Glycerol	12	N/A
Solvent	234	487
B-values (Å^2^)		
Protein (A/B/C/D/E/F)	36.2	30.7/43.9/36.9/38.9/35.6/40.0
Sulfate	N/A	68.0
Co/Cd/Cl	44.9/28.7/27.8	N/A
Glycerol	45.1	N/A
Solvent	38.8	33.3
r.m.s. deviation from ideality		
Bond lengths (Å)	0.013	0.004
Bond angles (deg.)	1.39	1.12
Ramachandran statistics (%)		
Most favoured	98.1	98.0
Allowed	1.9	2.0
Disallowed	0.0	0.0

Values in parentheses are for the highest resolution shell

^a^ R_merge_ = ∑_*hkl*_ ∑_*i*_ |I_*hkl*,*i—*_[I_*hkl*_]| / ∑_*hkl*_ ∑_*i*_ I_*hkl*,*i*_, where [I_*hkl*_] is the average of symmetry related observations of a unique reflection

^b^ R_work_ = ∑|F_obs_-F_calc_|/∑F_obs_, where F_obs_ and F_calc_ are the observed and the calculated structure factors, respectively

^c^ R_free_ is R using 5% of reflections randomly chosen and omitted from refinement

## Results

### Engineered deletion of the *Tg*AMA1 DII loop reveals a mature apical ligand binding groove

**Fig 1:**
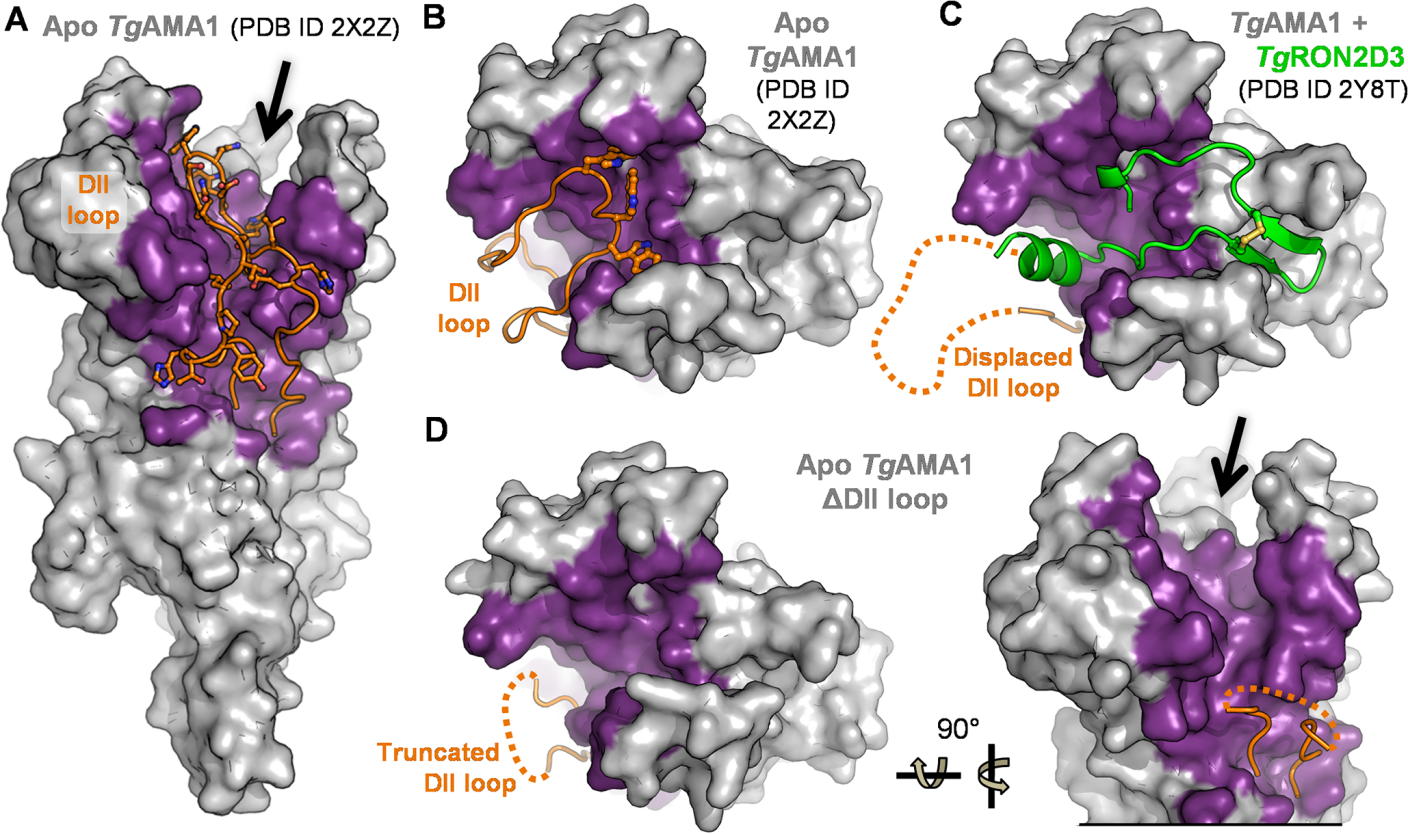
Engineered form of *Tg*AMA1 with a truncated DII loop presents a mature ligand binding groove. **(A)** Side view of apo *Tg*AMA1 (PDB ID: 2X2Z) displayed as a grey surface. Residues of *Tg*AMA1 DI and DII that interact with the DII loop are colored purple. The DII loop is shown as an orange cartoon, with residues that are displaced upon RON2 binding shown as ball-and-stick and colored by element. The arrow indicates the RON2 ligand binding groove. **(B)** Apical view of *Tg*AMA1, colored as in (A). The three Trp residues at the tip of the DII loop that anchor it into the apical surface are shown as sticks: W350, W353, W354. **(C)** Apical view of *Tg*AMA1 (colored as in (A)) complexed with *Tg*RON2 (green cartoon backbone, with disulfide bond shown in ball-and-stick) from PDB ID 2Y8T. The displaced region of the *Tg*AMA1 DII loop is indicated by a dotted orange line. **(D)** Apical (left) and side (right) view of *Tg*AMA1ΔDIIloop shown as a grey surface, with residues that correspond to DII loop coordinating residues of apo *Tg*AMA1 colored purple, and the truncated DII loop colored orange; the disordered Gly-Ser linker is shown as an orange dotted line. The arrow indicates the mature ligand binding groove presented in the absence of the DII loop.

*Toxoplasma gondii* AMA1 adopts a stacked three domain architecture, with Domain I (DI) membrane distal and Domain III (DIII) membrane proximal [24]. The apical surface is comprised of several loops from DI framing an apical groove, and a single extended loop from DII (DII loop) that packs against the side of DI and is stabilized by three tryptophan anchors buried into pockets on the *Tg*AMA1 apical surface (Fig 1A and 1B). Previous structural characterization of *Tg*AMA1 in complex with a RON2 peptide (*Tg*RON2D3) revealed that in order for RON2 to access the *Tg*AMA1 apical groove, the groove-occluding DII loop must be displaced (Fig 1C) [18]. To probe the role of the DII loop, we engineered a construct of *Tg*AMA1 with the twenty residue mobile portion of the DII loop replaced with a shortened Gly-Ser linker (*Tg*AMA1ΔDIIloop). This engineered construct of *Tg*AMA1 was produced in insect cells, purified, crystallized, and the apo crystal structure solved to 1.93 Å resolution. Superposition of *Tg*AMA1ΔDIIloop on RON2-bound *Tg*AMA1 resulted in a root mean square deviation (rmsd) of 0.70 Å over 365 Cα, indicating strong structural conservation of the protein core. Importantly, a comparison of the apical loops that frame the length of the ligand binding groove showed a maximum deviation of 0.91 Å with an average rmsd of 0.35 Å over 13 to 21 Cαs for the six DI loops indicating that minimal displacement of these loops in *Tg*AMA1ΔDIIloop is necessary for ligand coordination. Overall, truncation of the DII loop in *Tg*AMA1ΔDIIloop appears to faithfully mimic the displaced DII loop form of *Tg*AMA1 (Fig 1C) and thereby present a mature ligand binding groove (Fig 1D).

### *Tg*AMA1 is a more promiscuous receptor in the absence of the DII loop

**Fig 2:**
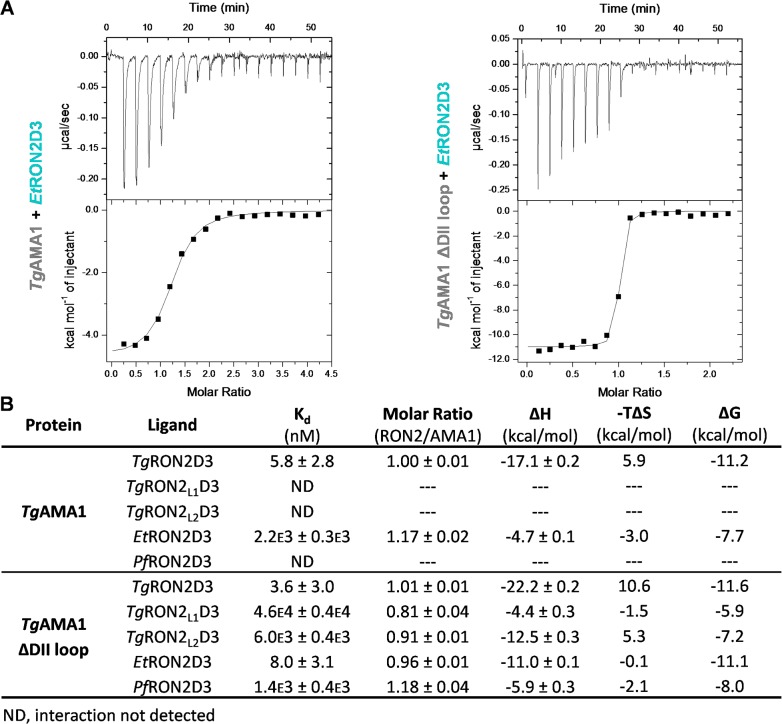
Isothermal titration calorimetry shows that the DII loop limits the reactivity of *Tg*AMA1. **(A)** Representative ITC thermograms for low affinity (left: *Et*RON2D3-TRX titrated into *Tg*AMA1) and high affinity (right: *Et*RON2D3-TRX titrated into *Tg*AMA1ΔDIIloop) complex formation. **(B)** Table of ITC results comparing binding affinity and thermodynamic parameters for *Tg*AMA1 and *Tg*AMA1ΔDIIloop binding to a panel of TRX-fused RON2D3 peptides at 25 °C.

To determine how the absence of the DII loop affected AMA1-RON2 complex formation, we used ITC to measure the solution binding characteristics of several RON2D3 peptides to *Tg*AMA1 or *Tg*AMA1ΔDIIloop (Fig 2A and 2B). *Tg*AMA1 bound to *Tg*RON2D3 with a low nanomolar affinity (5.8 nM or greater), consistent with previously published SPR measurements [18]. *Tg*AMA1 showed weak binding to *Et*RON2D3 (2.2 μM), while binding was not detectable (ND—estimated to be in millimolar range and therefore likely not relevant to anchoring the junction) for *Toxoplasma* RON2 paralogues (*Tg*RON2_L1_D3 and *Tg*RON2_L2_D3) or *Pf*RON2D3, consistent with previously established selectivity of *Tg*AMA1 for *Tg*RON2D3 [14, 15, 18]. Intriguingly, *Tg*AMA1ΔDIIloop displayed notably different binding profiles compared to *Tg*AMA1, aside from a similar affinity for the cognate ligand, *Tg*RON2D3 (Fig 2B). Most strikingly, *Et*RON2D3 bound approximately 275 fold tighter to *Tg*AMA1ΔDIIloop (8.0 nM or greater) relative to *Tg*AMA1 (2.2 μM). In addition, binding was detected between *Tg*AMA1ΔDIIloop and *Tg*RON2_L2_D3, *Tg*RON2_L1_D3 and *Pf*RON2D3 in the micromolar range (6.0, 46, and 1.4 μM, respectively) (Fig 2B). Despite the comparatively weaker affinities, each of these three interactions was saturable and showed a stoichiometry close to 1:1 consistent with genuine complex formation. Collectively, these results suggest that while the *Tg*AMA1 groove is fundamentally capable of interacting with all the RON2D3 peptides tested, the presence of the DII loop imparts remarkable selectivity.

### Structural basis of *Tg*AMA1ΔDIIloop-*Et*RON2D3 complex formation

**Fig 3:**
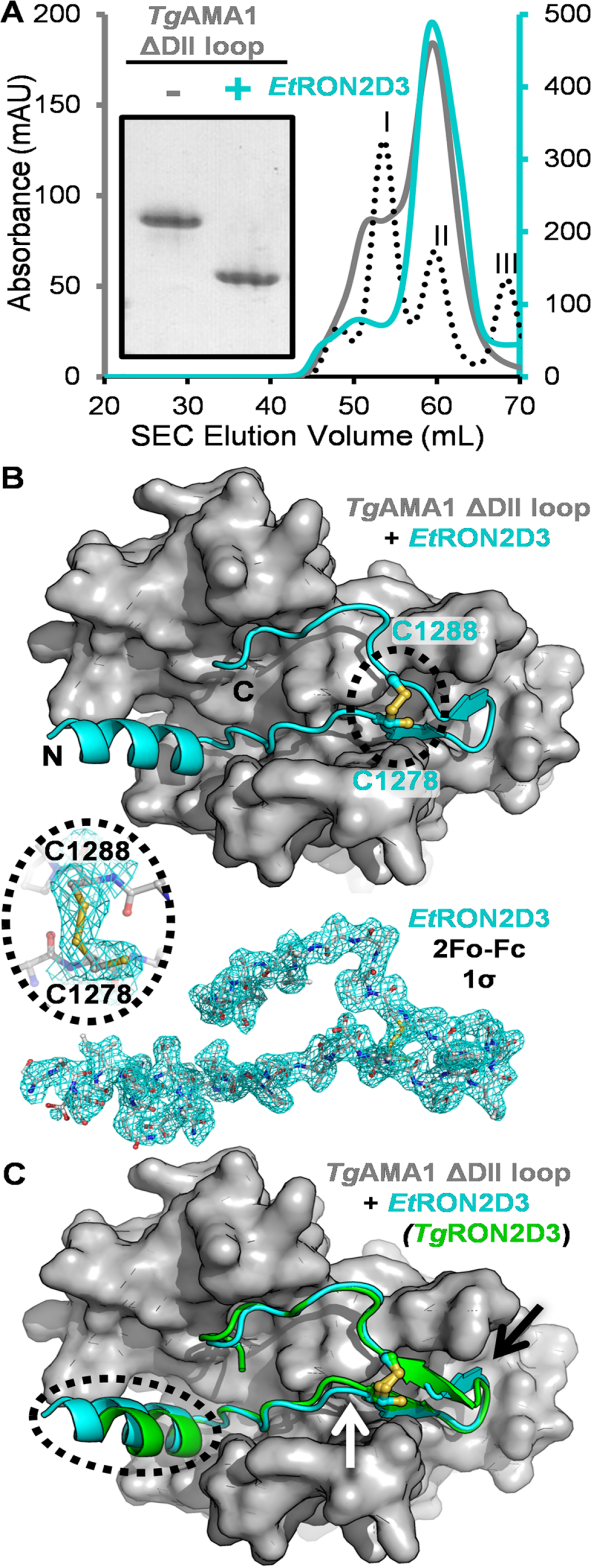
Characterization of a cross-genus AMA1-RON2 complex: *Tg*AMA1ΔDIIloop-*Et*RON2D3. **(A)** Size exclusion chromatograms of *Tg*AMA1ΔDIIloop with and without *Et*RON2D3 showing elution at the same volume. Globular molecular weight standards: I—75 kDa, II—43 kDa, III—29 kDa. Inset—gradient native PAGE of *Tg*AMA1ΔDIIloop without and with *Et*RON2D3 showing an effect on the migration of *Tg*AMA1ΔDIIloop in the presence of *Et*RON2D3. **(B)** Top—Apical view of *Tg*AMA1ΔDIIloop (grey surface) bound to *Et*RON2D3 (cyan cartoon backbone, disulfide and partially free cysteine shown as ball-and-stick and highlighted with black dotted oval). Bottom—Sigma-A weighted 2Fo-Fc electron density map contoured at 1.0 σ for *Et*RON2D3. Inset—zoom in on the electron density of the two Cys of *Et*RON2D3 chain F, which had the closest to 50% occupancy in each position for Cys1278. **(C)** Superposition of *Tg*RON2D3 on the *Tg*AMA1ΔDIIloop-*Et*RON2D3 complex. Different angles of the N-terminal helices are indicated by the dotted black oval; altered positioning of the *Et*RON2D3 coil leading up to the partially formed disulfide, white arrow; divergence of the cystine loop tip, black arrow.

The significant differential binding observed between *Et*RON2D3 and *Tg*AMA1 or *Tg*AMA1ΔDIIloop provided an experimentally tractable starting point from which to investigate the structural basis of how the DII loop governs selectivity. While *Tg*AMA1ΔDIIloop in the presence or absence of *Et*RON2D3 eluted in the same volume off the SEC column (Fig 3A), native PAGE analysis clearly showed an altered migration for the sample co-purified with *Et*RON2D3 (pI of 3.9) indicating that the peptide was retained by *Tg*AMA1ΔDIIloop throughout the purification process (Fig 3A, inset). The 2.20 Å resolution crystal structure of *Tg*AMA1ΔDIIloop in complex with *Et*RON2D3 showed clear electron density for the peptide seated in the apical groove (Fig 3B). Three complexes in the asymmetric unit showed little variation in overall structure (*Tg*AMA1ΔDIIloop: 0.32/0.60 Å rmsd over 392/387 Cα, chain A compared to C/E; *Et*RON2D3: 0.23/0.32 Å rmsd over 39/39 Cα, chain B compared to D/F), although an O-linked glycosylation on Thr425 of the DII-DIII linker was modelled only in chain A. Since *Et*RON2D3 chain D showed the lowest thermal motion (Table 1), the *Tg*AMA1ΔDIIloop chain C complex with *Et*RON2D3 chain D was used for further analyses unless otherwise noted.

*Et*RON2D3 bound throughout the apical groove of *Tg*AMA1ΔDIIloop in the expected conformation based on previous AMA1-RON2 co-structures [15, 18, 19], with the *Et*RON2D3 N-terminal helix seated in the area exposed by the absence of the *Tg*AMA1 DII loop, followed by a length of ordered coil, a beta-hairpin loop, and ordered C-terminal coil that extends back through the apical groove (Fig 3B). In contrast to previously characterized AMA1-RON2 complexes, the electron density surrounding the cysteine residues of *Et*RON2D3 (Cys1278 and Cys1288) revealed a population with only a partially formed disulfide bond. While the second cysteine (Cys1288) was appropriately positioned to form the disulfide, density based refinement of the occupancy for the first cysteine (Cys1278) showed 41 to 74% oriented away from the central axis of the disulfide bond (Fig 3B, inset). While the basis for this observation is unclear, it is possible that the less than ideal fit of *Et*RON2D3 into the *Tg*AMA1 groove may contribute to this anomaly.

### RON2 must engage both ends of the AMA1 groove to outcompete the DII loop

**Fig 4:**
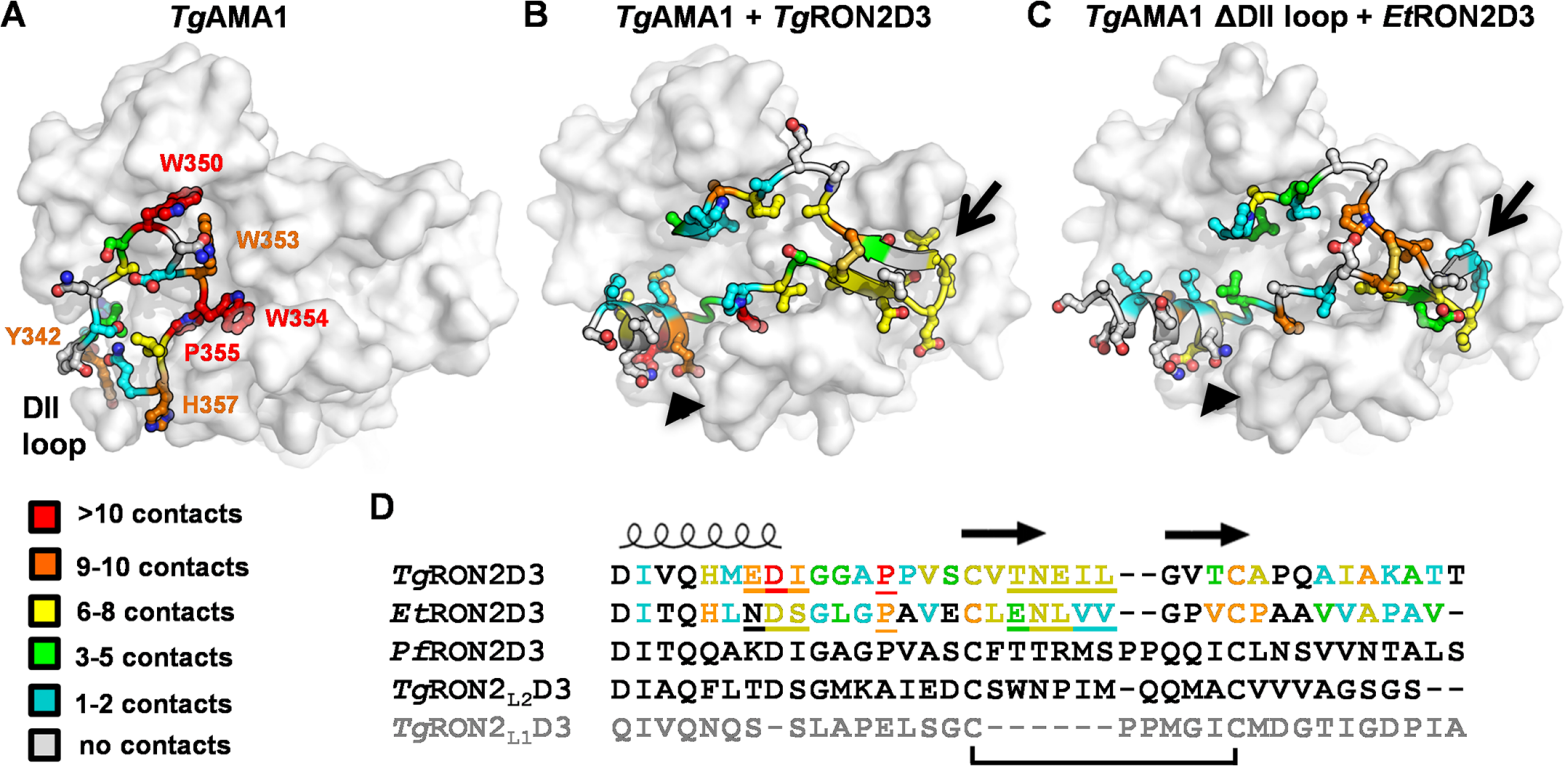
Interatomic contacts between *Tg*AMA1 and RON2D3 reveal high contact density areas likely required to outcompete the DII loop. **(A)** Heat map coloring scheme based on number of interatomic contacts from white (no contacts) to red (>10 contacts) shows that the DII loop (cartoon backbone with ball-and-stick sidechains) is anchored into the *Tg*AMA1 apical surface predominately through six residues, five of which are located in the apical groove (PDB ID: 2X2Z). **(B)** A similar analysis of contacts at the *Tg*AMA1-*Tg*RON2D3 interface (PDB ID: 2Y8T) shows that anchoring of the RON2 peptide occurs at the base of the N-terminal helix (black arrowhead), a proline in between the helix and disulfide bond, and the tip of the cystine loop (black arrow). **(C)** A contact heat map of the *Tg*AMA1ΔDIIloop-*Et*RON2D3 interface shows major reductions in the number of contacts formed between the *Tg*AMA1 apical groove and both the base of the RON2 helix (black arrowhead) and the tip of the cystine loop (black arrow). **(D)** Disulfide-anchored (black bar) sequence alignment of RON2D3 peptides used in this study, with the secondary structure representative of all peptides except the uncharacterized *Tg*RON2_L1_D3 (light grey) shown above, and *Tg*RON2D3/*Et*RON2D3 sequences colored on the interatomic contact heat map scale. Underlined residues correspond to anchored regions with highest interatomic contact density.

An initial comparison of the *Tg*AMA1-*Tg*RON2D3 and *Tg*AMA1ΔDIIloop-*Et*RON2D3 interfaces revealed similar overall buried surface areas (~3400 Å^2^ for each complex [35]), but an overlay of the *Tg*RON2D3 peptide on the *Tg*AMA1ΔDIIloop-*Et*RON2D3 complex shows key areas of divergence. Most importantly, the N-terminal helix adopts a different angle between the two peptides, the coil leading up to the first cysteine is restructured, and the tip of the cystine loop is shifted (Fig 3C). To investigate how these differences contribute to the inability of *Et*RON2D3 to outcompete the DII loop of *Tg*AMA1, we first examined the stabilizing hydrogen bond network between *Tg*RON2D3 or *Et*RON2D3 and the *Tg*AMA1 apical groove. Overall, the *Tg*AMA1ΔDIIloop-*Et*RON2D3 complex retains only sixteen of the twenty hydrogen bonds observed in the *Tg*AMA1-*Tg*RON2D3 complex [18], consistent with the less favorable enthalpy measured for *Tg*AMA1ΔDIIloop-*Et*RON2D3 by ITC (Fig 2B). Notably, the four hydrogen bonds that *Et*RON2D3 fails to form map to the N- and C-terminal regions of *Tg*RON2D3 that overlap with the hydrogen bond network of the DII loop in apo *Tg*AMA1 (Table 2) [35]. Specifically, the eight hydrogen bonds that must be broken to displace the DII loop from the apical groove are compensated for by the ten hydrogen bonds formed between *Tg*AMA1 and *Tg*RON2D3 in the overlapping region, contrasted against only six between *Tg*AMA1ΔDIIloop and *Et*RON2D3. We next assessed the number of interatomic contacts less than 3.9 Å formed between *Tg*AMA1 and each residue of the DII loop, *Tg*RON2D3, or *Et*RON2D3. The analysis of the DII loop revealed six key interaction points that must be disrupted in order to accommodate a ligand: Tyr342 forms a clamp near the base of the DII loop while Trp350, Trp353, Trp354, Pro355, and His357 all anchor the tip of the DII loop into the *Tg*AMA1 apical groove (Fig 4A). *Tg*RON2D3, which is capable of binding *Tg*AMA1 in the presence or absence of the DII loop, forms critical anchor points throughout the groove and in particular at the base of the N-terminal helix, the Pro residue between the helix and disulfide bond, and the five residues that form the tip of the cystine loop (Fig 4B). Importantly, these three regions correspond to the regions of altered topology between the two peptides (Fig 3C), and also correlate to regions with the largest number of contacts lost by *Et*RON2D3. In particular, three residues at the base of the N-terminal helix of *Tg*RON2D3 form extensive contacts to the *Tg*AMA1 groove, while the analogous residues of *Et*RON2D3 are positioned similarly but make one third the number of contacts (Fig 4C). The differences around the proline and the first cysteine are likely related to incomplete closure of the *Et*RON2D3 disulfide bond, and the Val-Val pair at the tip of the cystine loop is the only other region where consecutive residues of *Et*RON2D3 make substantially fewer interatomic contacts with *Tg*AMA1 compared to *Tg*RON2D3 (Fig 4D). Together, these analyses suggest that in order to maintain the DII loop in a displaced conformation, the ligand must engage anchor points in both the region exposed by displacement of the DII loop and at the opposite end of the AMA1 apical groove.

**Table 2:** Comparison of hydrogen bonds (< 3.5 Å or conserved) observed at the *Tg*AMA1ΔDIIloop-*Et*RON2D3 and *Tg*AMA1-*Tg*RON2D3 interfaces.

*Et*RON2D3	*Tg*AMA1 ΔDII loop	Distance (Å)	*Tg*RON2D3	*Tg*AMA1	Distance (Å)
			Glu1303 [Oε2]	Arg111 [N]	3.46
			Glu1303 [Oε1]	Gln361 [Nε2]	2.55
Asp1269 [Oδ1]	Gln361 [Nε2]	2.83	Asp1304 [Oδ1]	Gln361 [Nε2]	2.75
Asp1269 [O]	Arg111 [NH1]	2.87	Asp1304 [O]	Arg111 [NH1]	2.98
**Gly1271 [O]**	**Met233 [N]**	**3.74**	**Gly1306 [O]**	**Met233 [N]**	**3.65**
Val1276 [N]	Tyr230 [OH]	3.57	Val1311 [N]	Tyr230 [OH]	3.54
Val1276 [O]	Tyr230 [OH]	3.42	Val1311 [O]	Tyr230 [OH]	2.56
**Cys1278 [O]**	**Met204 [N]**	**3.04**	**Cys1313 [O]**	**Met204 [N]**	**3.06**
**Glu1280 [N]**	**Val202 [O]**	**2.96**	**Thr1315 [N]**	**Val202 [O]**	**2.90**
**Glu1280 [O]**	**Val202 [N]**	**3.06**	**Thr1315 [O]**	**Val202 [N]**	**2.85**
Asn1281 [Nδ2]	Phe197 [O]	3.58	Asn1316 [Nδ2]	Phe197 [O]	3.47
Asn1281 [Nδ2]	Lys200 [O]	3.58	Asn1316 [Nδ2]	Lys200 [O]	3.70
Asn1281 [Nδ2]	Thr201 [Oγ1]	2.63	Asn1316 [Nδ2]	Thr201 [Oγ1]	2.76
**Leu1282 [N]**	**Lys200 [O]**	**3.27**	**Glu1317 [N]**	**Lys200 [O]**	**2.99**
**Cys1288 [N]**	**Val164 [O]**	**3.04**	**Cys1323 [N]**	**Val164 [O]**	**2.92**
**Cys1288 [O]**	**Val164 [N]**	**2.77**	**Cys1323 [O]**	**Val164 [N]**	**2.94**
**Val1292 [O]**	**Glu145 [N]**	**2.85**	**Ala1327 [O]**	**Glu145 [N]**	**2.87**
**Ala1294 [N]**	**Pro143 [O]**	**3.12**	**Ala1329 [N]**	**Pro143 [O]**	**2.97**
			Lys1330 [N]	Glu145 [Oε1]	3.16
			Ala1331 [O]	Trp253 [Nε1]	3.35

Sidechain independent interactions are bolded.

## Discussion

High affinity AMA-RON2 complexes anchor the junction between apicomplexan parasites and host cells and play an important role in invasion [10, 14, 36]. Functional and biophysical dissection of cognate AMA-RON2 pairs has led to detailed insight into how these complexes support invasion, which has in turn helped guide the development of novel therapeutic intervention strategies [15, 18, 19, 37, 38]. Notably, the AMA1-RON2 complex was recently shown to be significantly more immuno-protective than AMA1 alone for malaria [39, 40], and the development of pharmacophore models describing the AMA1 ligand binding groove have led to *in silico* docking experiments and corresponding proof of principle invasion inhibition studies [17, 41–44]. To further probe the complexities of this sophisticated invasion complex, we have interrogated the role of the AMA1 DII loop, a key substructure that must be displaced for RON2 binding [15, 18, 19, 23, 24].

Initially, we engineered a form of *T*. *gondii* AMA1 where the flexible region of the DII loop was replaced with a short Gly-Ser linker (Fig 1). Solution binding analyses using a diverse panel of RON2 peptides revealed a critical role for the DII loop in regulating *Tg*AMA1 selectivity. Notably, *Tg*AMA1 bound both *Tg*RON2D3 and *Et*RON2D3 but only the former with high affinity, while *Tg*AMA1ΔDIIloop showed moderate binding to each tested RON2D3 and bound both *Tg*RON2D3 and *Et*RON2D3 with high affinity (Fig 2). To establish the molecular basis for the observed differential binding, we determined the structure of *Tg*AMA1ΔDIIloop in complex with *Et*RON2D3. Notably, this is the first cross-genus AMA-RON2 pair to be structurally characterized (Fig 3). A comparison of contacts in the *Tg*AMA1-*Tg*RON2D3 and *Tg*AMA1ΔDIIloop-*Et*RON2D3 structures enabled the identification of key anchor points in the AMA1 groove exploited to outcompete the DII loop and bind *Tg*AMA1 with high affinity (Fig 4). Intriguingly, the anchor points identified at the base of the RON2 N-terminal helix and the tip of the cystine loop correlate with the regions in which the most successful linear peptide inhibitor of the AMA1-RON2 interaction to date, peptide R1, binds *Pf*AMA1 [19, 45]. These data further support a role for the DII loop in tuning AMA1 selectivity. The detailed mechanism by which the DII loop achieves the required selectivity, however, remains to be determined and will likely require measuring pre-equilibrium kinetics of association and dissociation between RON2 and both native and ΔDIIloop forms of AMA1 from *T*. *gondii* and *P*. *falciparum*.

The biological implications of truncating the DII loop have proven difficult to assess. However, since the presence of the AMA1 DII loop results in a more selective receptor, this substructure may act as a structural gatekeeper to restrict formation of unproductive AMA1 complexes. Thus, a more discriminating AMA1 apical groove would ensure the parasite-host cell junction is competent for invasion because only a cognate RON2 would bind with sufficient affinity to outcompete the DII loop. This gatekeeping function may ultimately serve as a molecular switch to signal the parasite that a functional junction has been formed and invasion can proceed. It follows that an apicomplexan parasite expressing the truncated DII loop form of AMA1 might experience more abortive invasion events resulting from unproductive, non-RON2 based AMA1 complexes that are not linked to junction integrity. The potential for AMA1 to form non-RON2 based complexes has been proposed [46–50], particularly in *Plasmodium*, and it is even suggested that a complex between AMA1 and a host surface protein may support a pre-invasion step [51]. In this case the parasite would use AMA1 as a simple adhesin without activating the motor system to drive invasion. While no host cell ligands have been identified that bind the apical groove of AMA1, weak affinities that rely on receptor clustering in a cellular context may have limited their identification. Using AMA1 ΔDIIloop truncation constructs in pulldown assays with host lysates may overcome this limitation as we have shown in the case of *Tg*AMA1 that the ΔDIIloop construct displays a more permissive binding profile. Furthermore, engineering parasites to express ΔDIIloop constructs of AMA1 will enable the investigation of potential DII loop dependant signalling events associated with high affinity AMA1-RON2 binary complex assembly and moving junction formation.

## Conclusions

The solution binding studies of several RON2 peptides with *Tg*AMA1 and *Tg*AMA1ΔDIIloop reveal a previously underappreciated role for the DII loop in selectively filtering out ligands otherwise capable of binding in the AMA1 apical groove. Companion structural studies offer important insight into molecular recognition thresholds that likely need to be achieved by ligands able to outcompete the DII loop and form high affinity complexes capable of anchoring the moving junction during invasion of the host cell. While the biological implications of apicomplexan AMA1 DII loop conformational changes remain an intriguing topic for future studies, these results provide a molecular basis for understanding AMA1 selectivity, which can support the development of novel therapies targeting the important AMA1-RON2 invasion complexes.
